# A New Mass Mortality of Juvenile *Protoceratops* and Size-Segregated Aggregation Behaviour in Juvenile Non-Avian Dinosaurs

**DOI:** 10.1371/journal.pone.0113306

**Published:** 2014-11-26

**Authors:** David W. E. Hone, Andrew A. Farke, Mahito Watabe, Suzuki Shigeru, Khishigjav Tsogtbaatar

**Affiliations:** 1 School of Earth Sciences, University of Bristol, Bristol, United Kingdom; 2 School of Biological & Chemical Sciences, Queen Mary University of London, London, United Kingdom; 3 Raymond M. Alf Museum of Paleontology, Claremont, California, United States of America; 4 Hayashibara Museum of Natural Sciences, Okayama, Japan; 5 Paleontological Center, Mongolian Academy of Sciences, Ulaanbaatar, Mongolia; University of Pennsylvania, United States of America

## Abstract

**Background:**

Monodominant bonebeds are a relatively common occurrence for non-avian dinosaurs, and have been used to infer associative, and potentially genuinely social, behavior. Previously known assemblages are characterized as either mixed size-classes (juvenile and adult-sized specimens together) or single size-classes of individuals (only juveniles or only adult-sized individuals within the assemblage). In the latter case, it is generally unknown if these kinds of size-segregated aggregations characterize only a particular size stage or represent aggregations that happened at all size stages. Ceratopsians (“horned dinosaurs”) are known from both types of assemblages.

**Methods/Principal Findings:**

Here we describe a new specimen of the ceratopsian dinosaur *Protoceratops andrewsi*, Granger and Gregory 1923 from Mongolia representing an aggregation of four mid-sized juvenile animals. In conjunction with existing specimens of groups of *P. andrewsi* that includes size-clustered aggregations of young juveniles and adult-sized specimens, this new material provides evidence for some degree of size-clustered aggregation behaviour in *Protoceratops* throughout ontogeny. This continuity of size-segregated (and presumably age-clustered) aggregation is previously undocumented in non-avian dinosaurs.

**Conclusions:**

The juvenile group fills a key gap in the available information on aggregations in younger ceratopsians. Although we support the general hypothesis that many non-avian dinosaurs were gregarious and even social animals, we caution that evidence for sociality has been overstated and advocate a more conservative interpretation of some data of ‘sociality’ in dinosaurs.

## Introduction

The fossil record of non-avian dinosaurs (hereafter simply ‘dinosaurs’) includes numerous concentrations of individuals that suggest aggregations formed during life. Examples are known for both major clades of dinosaurs (Ornithischia and Saurischia), with data from trackways e.g. [1]–[2], nesting sites e.g. [3] and bonebeds resulting from mass mortality events confirm the presence of in-life aggregations e.g. [4]–[7]. Although some of these concentrations may reflect non-biological taphonomic processes, drought assemblages or predator traps rather than genuine group behavior e.g. [8]–[9], there is overall little doubt that at least some dinosaurs occasionally resided in groups, potentially for significant durations.

Notably, non-adult individuals dominate many dinosaur aggregations (see [10] and references therein0, suggesting the possibility of a level of gregariousness in juveniles not necessarily seen in adults [11]. With the exception of hatchlings in nests, juveniles may be accompanied by adults e.g. [4], [12]–[15], in aggregations composed solely of juveniles or subadults of different sizes suggestive of multiple age classes e.g. [6]–[7], [16]–[17], or in aggregations composed entirely of juveniles of similar sizes (here termed “size-segregated aggregations” [5], [11], [18]–[20]). This suggests the mode of juvenile gregariousness varied both ontogenetically and phylogenetically.

Ceratopsians, or horned dinosaurs, present some of the most extensively documented occurrences of monodominant (comprised mostly of one taxon) and monospecific (composed exclusively of one taxon) fossil assemblages. Nearly all of these host juvenile specimens, and many are juvenile-dominated. Examples have been described for the basal ceratopsian *Psittacosaurus* [7], [21]–[22], the non-ceratopsid neoceratopsians *Prenoceratops* [23], *Protoceratops* [20], and *Zuniceratops* [24], and multiple species of ceratopsids (reviewed in [25], [26]). Coupled with detailed investigations of geology, taphonomy, anatomy, and histology, a broad picture of the phylogenetic and environmental distributions of group behavior is now possible. However, little information is available on how these aggregations may have formed or changed through the lifespan of individuals within a species. To date, there has been no evidence of size-segregated (and thus presumably age-segregated) aggregations for all ontogenetic stages of a taxon.

Here, we describe an aggregation of four closely associated juvenile *Protoceratops* and a pair of subadults of the same genus from the Djadokhta Formation of the Late Cretaceous, Mongolia. *Protoceratops*, a ceratopsian characterized by its thin and broad bony frill and nasal bump that develop through ontogeny, is one of the most abundant ceratopsians in Mongolia. The taxon is represented by hundreds of specimens from all ontogenetic stages [14]. These fossils allow a detailed understanding of ontogeny, possible sexual dimorphism, and behavior in an extinct organism. A presumed nest of 15 juvenile *Protoceratops* was described previously [20], but those individuals are much smaller than those described here, and aggregations of adult-sized individuals are also known [27]. Thus, these new finds bring important information on the ontogeny of fossil aggregations in *Protoceratops*, and the broadest documented occurrence of size-segregated group living through all stages of ontogeny in a non-ceratopsid neoceratopsian. We also discuss the aggregation behaviour of non-adults and their importance in dinosaurian ecology.

## Description

### Ontogenetic Definitions

Historically, ontogenetic classes (hatchling, juvenile, subadult, adult) in *Protoceratops* and other ceratopsian dinosaurs have been inferred by body size and the development of cranial features such as the frill and nasal ornamentation [28]–[30]. Osteohistological characters are increasingly used to determine the ontogenetic classes of non-avian dinosaurs [31]–[32], and a particularly important finding is that organisms of relatively large size have not necessarily terminated growth; i.e., developed an external fundamental system (EFS) within the bones. This is further highlighted by the fact that some taxonomically diagnostic features do not appear until comparatively large body size [33]–[34], and that even “adult” morphologies are modified late in growth [35]. Furthermore, at least some non-avian dinosaurs apparently reached sexual maturity long before reaching terminal body size or somatic maturity [36]–[38]. The result is a quagmire of varying definitions for ontogenetic stages and ontogenetic assignments across different publications even for single specimens. We note that considerably more study is needed to develop biologically grounded and reliable definitions for ontogenetic stages in non-avian dinosaurs, particularly given the current scarcity of work linking osteohistology, morphology, and life stages in extant taxa. Pending this sort of synthetic work, we will briefly lay out the criteria used for assigning ontogenetic stages here.

Preliminary analyses of *Protoceratops* suggest that the osteohistologically oldest specimens (i.e., those with an EFS) also have the most extreme development of cranial features, but that a slow-down of growth (in other dinosaurs associated with sexual maturity [37]) occurred well before this [39]–[40]. Pending full publication of these results, and in light of the caution required in age assessments, we adopt the following terminology for the discussion here. Both cranial morphology and overall skeletal element size are used to generate skeletal age classes here, using criteria modified from Brown and Schlaikjer [28] and Handa et al. [30]. These criteria are briefly summarized here, but the previous literature should be seen for full definitions.

Juveniles: (“young stage” of [30], partially equivalent to the “young” stage and also including the “very immature” and “immature” individuals of [28]) are the smallest in size and with the least development of the frill, frontoparietal depression, and nasal ornamentation, along with the lack of an epijugal. These individuals are less than 30 percent of adult size (linear measures for major elements e.g. femur). Additional criteria pertaining to this stage are outlined in Handa et al. [30].

Subadults: (first defined by Handa et al. [30], partially equivalent to the “young” and “young adult” stages of Brown and Schlaikjer [28]) shown a mix of juvenile and adult features, particularly in the poor development of the nasal ornamentation, combined with moderate expansion of the frill, initial ossification of the epijugal, and shape of the parietal fenestrae, among other features. These individuals are, unsurprisingly, intermediate in size between juveniles and adults (between ∼30 to 70 percent maximum adult size).

Adults: (broadly similar to the definitions in [30] and [28]) show strong expansion of the frill and strong development of a frontoparietal depression, at least moderate development of nasal ornamentation, rugose postorbitals, and the largest body sizes in the sample.

We note, of course, that these categories are necessarily imprecise and at present unassociated with osteohistological data. Owing to the nature of the specimens within our study, size is the most easily assessed variable. Regardless of any future changes to ontogenetic assessment in *Protoceratops*, our observations concern specimens spanning nearly the entire size range (and thus presumably ontogenetic range) of *Protoceratops*.

### Locality Information

**Figure 1:**
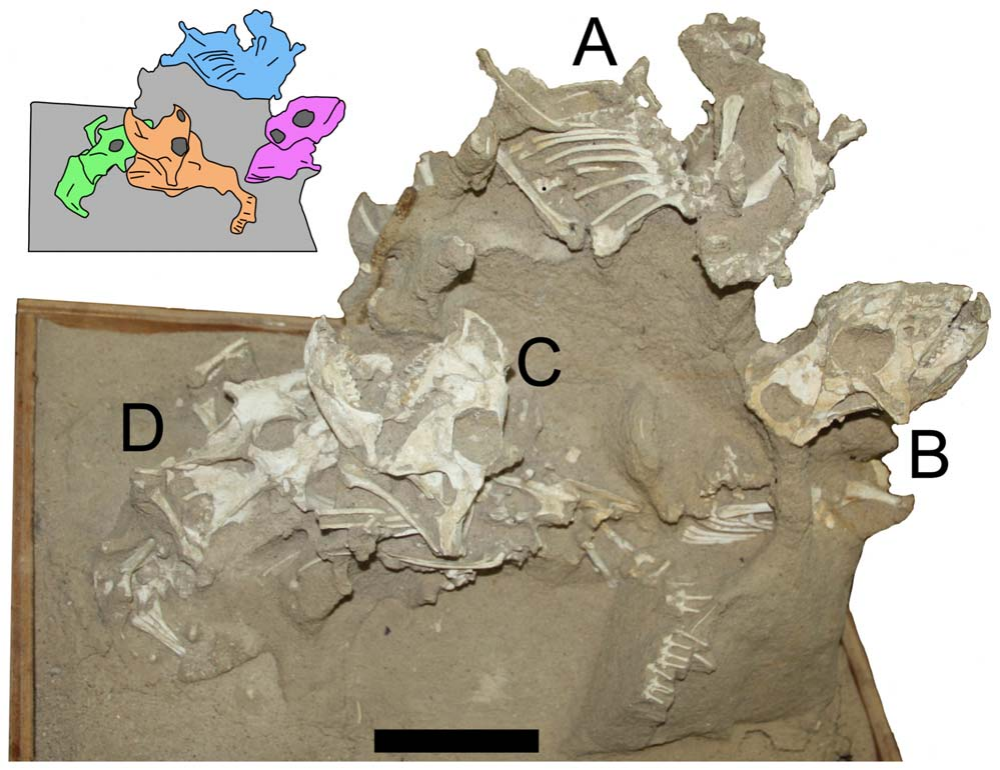
Specimen MPC-D *Protoceratops* labelled A–D. 100/526 of four juvenile Inset shows the division between the specimens. Scale bar is 100 mm.

The group of juveniles (MPC-D 100/526 – Figure 1 Mongolian Paleontological Center, Mongolian Academy of Sciences, Mongolia) was discovered and excavated in 1994 from the lower level of the aeolian sandstone sequence at the western part of the Tugrikin Shire locality, Central Gobi region, Mongolia (Field Permit: Paleontological Center, Mongolian Academy of Sciences, Ulaanbaatar, 14201, Mongolia). The stratigraphic level of the specimen is ca. 1.5 m below the zone of inter-dune that has wide lateral extension (nearly 300 west-east direction) with 1 to 2 m of thickness, consisting of massive (structureless) and sometimes of horizontally stratified sands, in the large scaled sand dune (erg) environment of the Djadokhta area. The thick sand parts with large-scaled cross-stratification are widely developed above and below this horizontal zone. Similar lithology and stratigraphy as an intedune sediment had been shown by Jerzykiewicz et al. [27] at the Djadokhta dinosaur locality Bayan Mandahu in Inner Mongolia. The erg environments that dinosaurs once occupied in this locality was elucidated by Fastovsky et al. [41] in their study of eolian fossil-bearing Cretaceous rocks in Mongolia.

**Figure 2:**
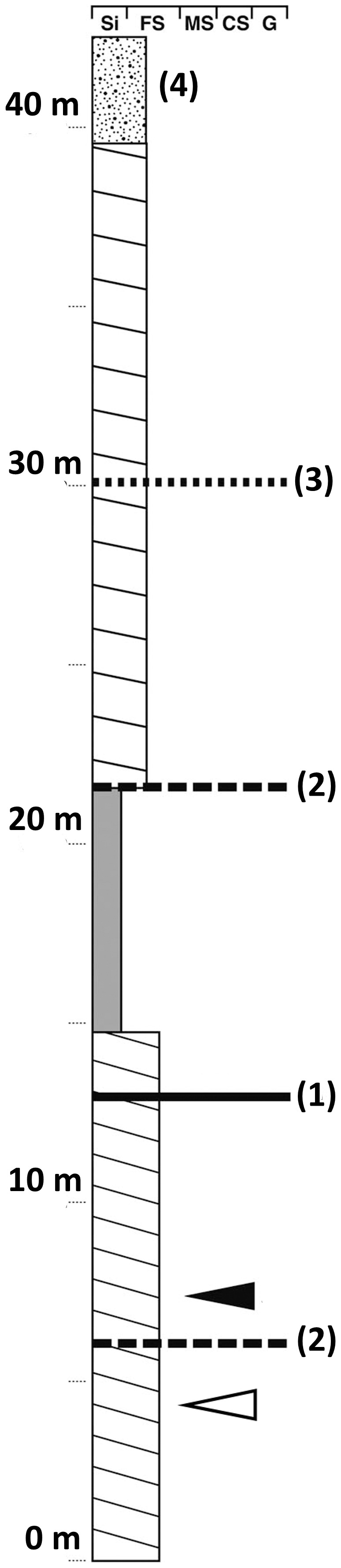
Geological column of Tugrikin Shire eolian beds. 1. First order boundary of eolian deposits. 2. Second order boundary. 3. Third order boundary. 4. Quaternary eolian beds. The horizon for this specimen is indicated by the white arrow, and the black arrow refers to the horizon that yielded the specimen on young juveniles described in [20].

Specimen MPC-D 100/526 was excavated from the aeolian sand bed as a single block containing all four individuals. The geological section (thickness of ca. 6 m) below than the specimen's level bears foreset beds of aeolian dune tilting to the northeast. The second specimen, a pair of subadult animals (MPC-D 100/534 - Mongolian Paleontological Center, Mongolian Academy of Sciences, Mongolia) was discovered at the central area of Tugrikin Shire in 1989 at roughly the same stratigraphic level as the juvenile aggregation MPC-D 100/526. The stratigraphic level of those two associations is slightly lower than that which yielded an aggregation of infant *Protoceratops* individuals [20] (Figure 2).

The aeolian sandstone beds have yielded many dinosaurs specimens including *Protoceratops*, *Velociraptor*, alvarezsaurs, and birds. The eolian deposits of the Tugrikin Shire locality are in part the result of re-working during the Cretaceous. This re-working (erosion of already deposited sands, and deposition of those eroded sands in different places) means that the skeletons of *Protoceratops* and other vertebrates had been eroded in the Cretaceous, perhaps a short time after their initial burial, and were destroyed on the surface. Isolated skulls with lower jaws, fully articulated caudal vertebrae, and postcranial skeletons without skulls are often found from these beds (MW pers obs.). The incompleteness of these skeletons are caused by contemporaneous post-depositional erosion in the Cretaceous, however such activities should not greatly affect the relative positions of the skeletons of individuals relative to one another and specimens subjected to significant post-depositional erosion would be expected to show a high degree of disarticulation, which is not seen here.

### Specimen MPC-D 100/526

As detailed anatomical descriptions of *Protoceratops* are already in the literature [28], [30], [42]–[43], we restrict our descriptions to taxonomically and ontogenetically informative characters and general comments about the preservation and orientation of the individuals of each specimen.

**Figure 3:**
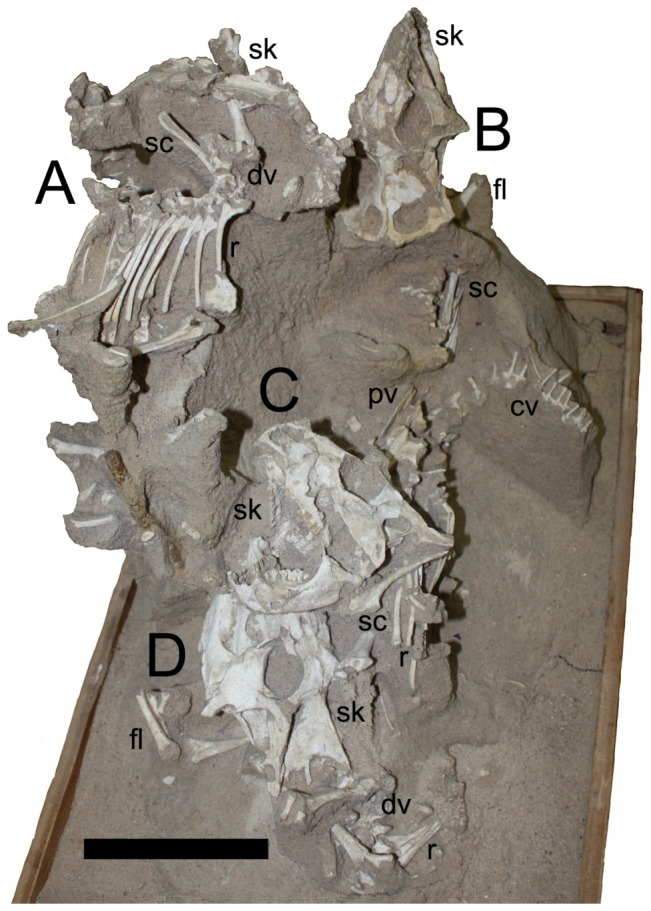
Left hand side view of specimen MPC-D 100/526 of four juvenile *Protoceratops*. Scale bar is 100 mm.

**Figure 4:**
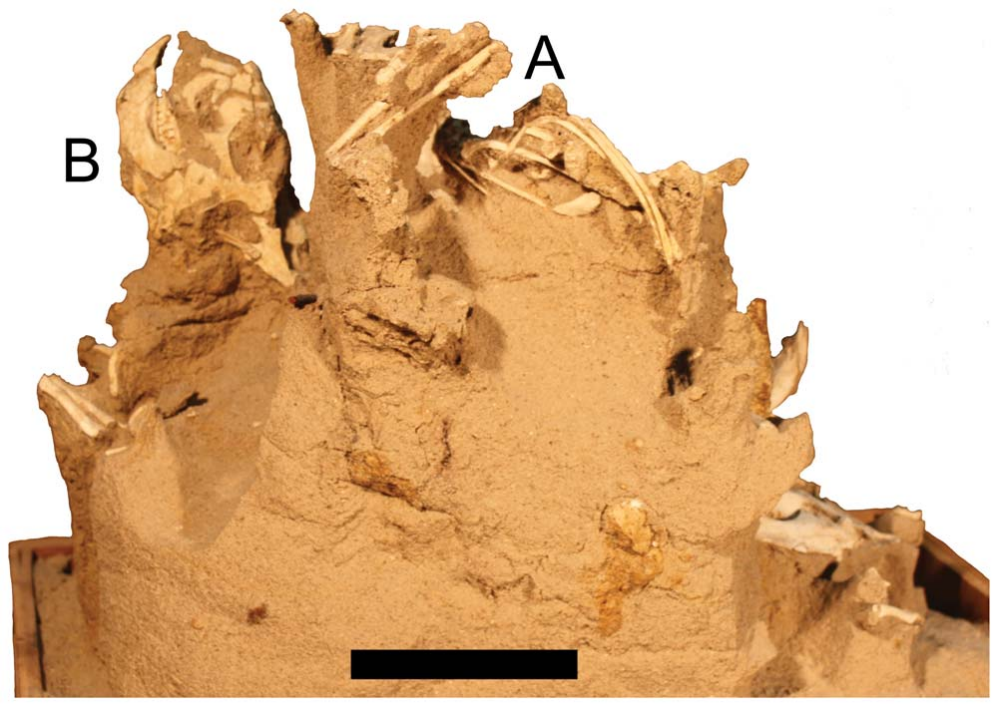
Rear view of MPC-D 100/526 of four juvenile *Protoceratops*. Scale bar is 100 mm.

**Figure 5:**
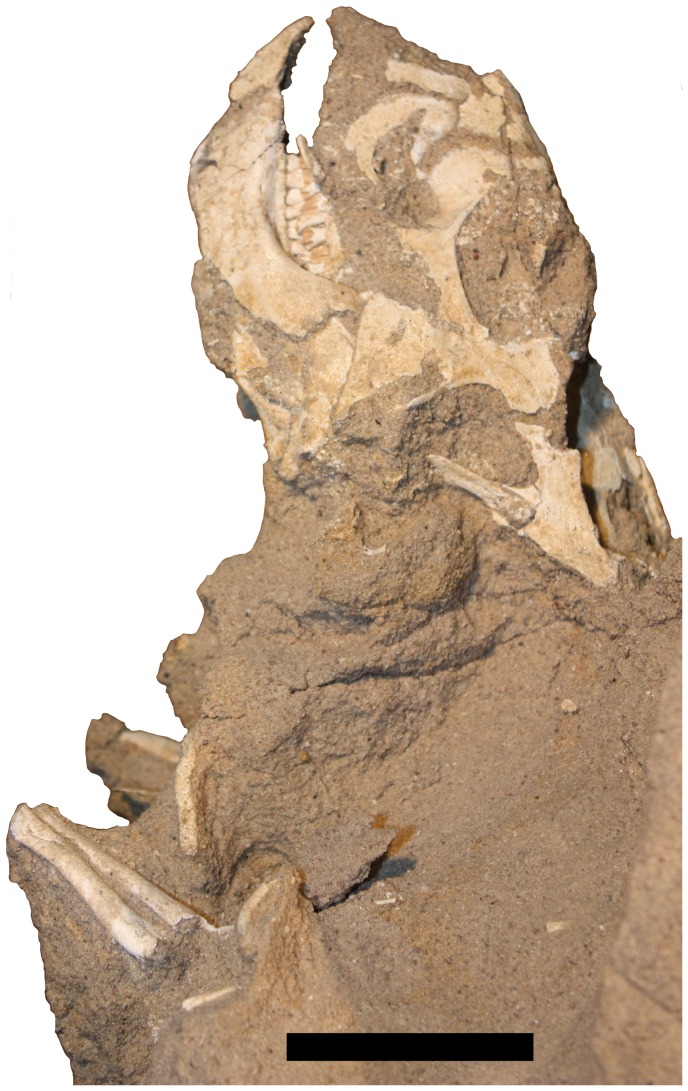
Individual B of MPC-D 100/526. Scale bar is 50 mm.

**Figure 6:**
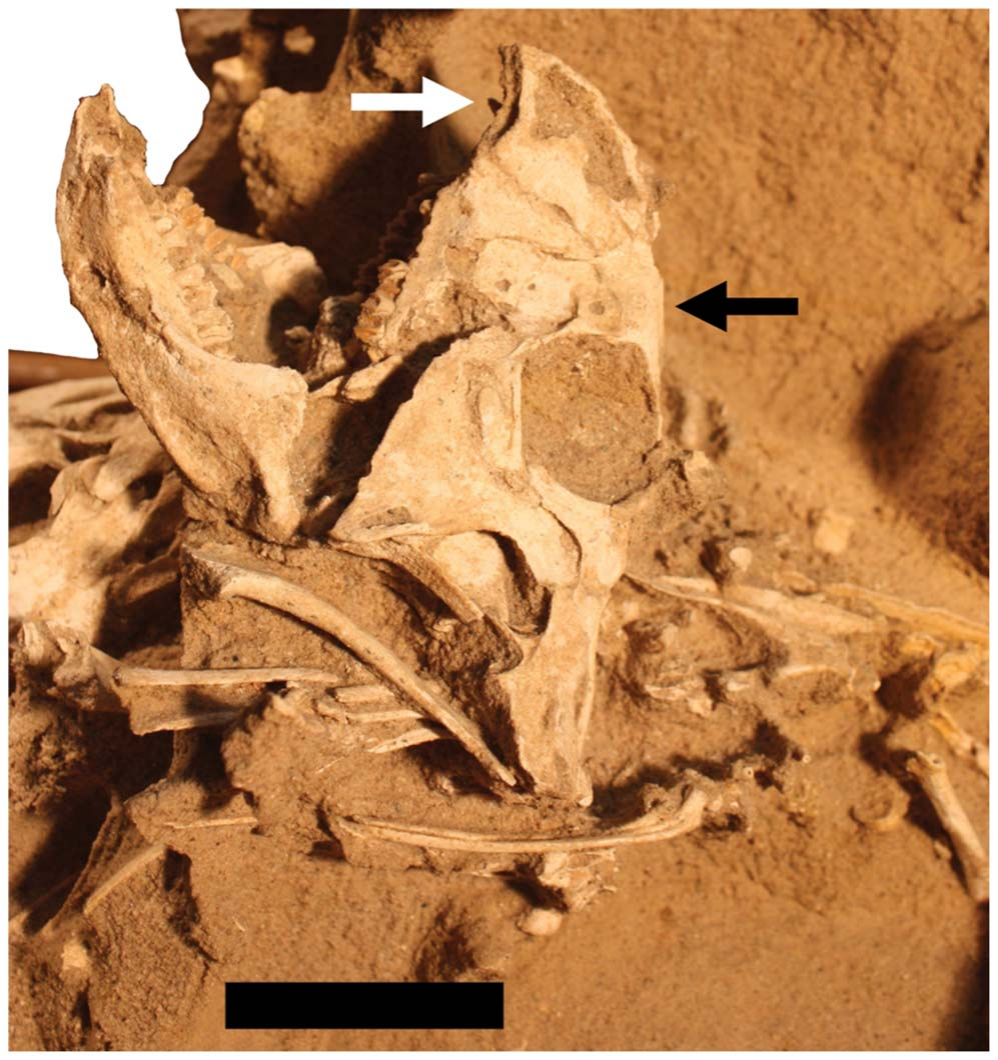
Skull of individual C of MPC-D 100/526. The white arrow indicates the premaxillary tooth that helps diagnose the specimen as *P. andrewsi*, and the black arrow points to damage to the skull from boring insect larvae. Scale bar is 50 mm.

Specimen MPC-D 100/526 is a large block of sandstone measuring 50 cm by 30 cm across the base and some 40 cm high (not including additional supporting material below the exposed area), containing the remains of four juvenile *Protoceratops andrewsi* (See Figures 1, 3–6). The individual animals are designated A-D, with the uppermost specimen being A and lowest one D. The matrix is uniform and consists of fine-grained, light coloured sand. The multiple individuals are lying at different angles to one another and the block as a whole, so relative positions are difficult to describe. As such, we use the terms left, right, front and back, to refer to relative positions within the block as a whole as seen in Figure 1.

Specimen A suffered significant erosion to the skull, and the postcranium is partially disarticulated. Specimens B-D are in near-natural articulation, although some elements settled a little post-depositionally. A is positioned largely horizontally, whereas B-D are positioned towards the vertical axis with the heads up. Aside from recent erosion, the bones are in generally excellent condition and are well preserved. The block underwent extensive laboratory preparation at the HMNS (Hayashibara Museum of Natural Sciences, Setouchi-Shi, Japan; now returned to Mongolian Paleontological Center, Mongolian Academy of Sciences, Mongolia) to expose as many elements as possible, but some areas were left covered in order to maintain the integrity of the piece as a whole. Thus, the completeness of B-D is inferred rather than demonstrated. The slight disarticulation and vertical displacement of some elements is ascribed to the settling of the sand after death and probably following decay of soft tissues. Insect larvae and burrowing animals may be responsible for at least some damage (e.g. holes bored into the left lacrimal and left anterior dentary of C – see Figure 6, or the articular ends of some longbones) and possibly also the disarticulation [27], [44]–[45]. Nonetheless, a lack of bite marks, shed teeth, and disarticulation rules out scavenging by vertebrates.

Individual A (Figure 1). This individual experienced extensive recent erosion prior to discovery, and some disarticulation (likely pre-fossilisation). The badly eroded skull faces towards the front of the block. Some indeterminate skull parts are preserved, as well as some elements that may be cervical vertebrae. At least ten dorsal vertebrae are present, with most of the dorsal ribs preserved in articulation. Eight dorsal ribs are in articulation on the right side of the specimen (plus an additional disarticulated rib) and seven are in articulation on the left. At least five caudal vertebrae are preserved in near normal articulation.

Each scapulacoracoid is preserved in association, but both pairs lie below the dorsal vertebral series, and the right elements lie with their medial faces uppermost. The left humerus lies under the remnants of the skull. The right arm as a whole is largely in articulation, though the radius and ulna lie as if the right humerus was lost between the right scapula and coracoid. Parts of the right leg lie below, but also behind, the dorsal series. The left tibia and fibula and some possible phalanges lie behind and underneath the left side of the skull.

These disarticulations and moved bones are inferred to be the result of the partial collapse or slipping of the enclosing sediment prior to consolidation and by extension prior to fossilisation. In each case (here and for specimens B–D), the movement of elements is vertical, rather than laterally or with random scattering.

Individual B (Figure 5). Only the anterior part of this individual is exposed on the right of the block. The head points up, whereas the postcranium lies horizontally towards the left. A near-complete cranium and articulated mandible are exposed below A and on the far right hand side of the specimen. The right surfaces of the dorsal vertebrae are in poor condition but preserved in articulation with a series of seven dorsal ribs. The right scapula and coracoid and an articulated humerus are visible on the right side of the animal, and on the left, a partial humerus and the radius and ulna are exposed.

Individual C (Figure 6). This individual is in the best condition and, as exposed, is the most complete of the four. As with B, the head faces upwards while the body in orientated horizontally, though in this case it lies with the head to the left of the block and the postcranium running left to right. It is positioned at a level just below that of B. The skull is complete and articulated with the mandible, though as preserved the mouth is clearly open. At least one premaxillary tooth is visible in the specimen as currently prepared. Several disarticulated dorsal vertebrae are visible, although five dorsal ribs lie as if articulated with what would have been the original position of the series. Both ilia are exposed, though these are damaged, and the tips of the neural spines of the sacral vertebrae are exposed between them. Thirteen caudal vertebrae are exposed and are nearly all articulated in a series. The midshafts of both femora are visible and appear to be in articulation with the pelvic region, and the loss of the femoral heads may be due to the action of insect larvae [44].

Individual D (Figure 3). Little of this individual is visible, because it lies almost directly below Individual C. The head lies to the left of the block and faces to the rear of the block with most of the body lying under the cranium of C. The skull is nearly complete and articulated with the mandible. A partial dorsal series exposed including four articulated dorsal ribs on the animal's right side. The left scapula and coracoid, left humerus, radius, ulna and some parts of the manus are visible and in articulation.

### Identification

Several features identify the specimens as derived neoceratopsians within Coronosauria (the clade including Protoceratopsidae and Ceratopsidae), particularly the broad, elongate, and fenestrate parietosquamosal frill [43]. Although the specimens are juveniles, and hence lack specific autapomorphies for *P. andrewsi* [30], they can be assigned to *Protoceratops* cf. *P. andrewsi* based on a combination of features. The specimens differ from *Bagaceratops rozhdestvenskyi* in the absence of an accessory antorbital fenestra and differ from *B. rozhdestvenskyi* and *Protoceratops hellinkorhinus* in the occurrence of premaxillary teeth [20], [30], [46] (Figure 6); both features are consistent with *Protoceratops andrewsi*, which is abundant at the locality. The dentary in the juvenile specimens here is relatively straight rather than bowed (more similar to adult *B. rozhdestvenski* than *Protoceratops* spp.), but bowing develops ontogenetically in *P. andrewsi* [28], so the feature is probably not of taxonomic significance.

#### Range of sizes

Owing to the articulation and preparation of the material, and the poor preservation of articular ends of many longbones, few elements can be easily measured or compared between specimens. Nonetheless, the measurements that can be taken show that the four specimens are all of similar size (see Table 1).

**Table 1:** Lengths of various parts of individuals A–D from MPC-D 100/526.

Measurement	A	B	C	D
Total length from tip of snout to middle of frill (measured along top of skull)	-	139	143*	148
Height of orbit at midpoint	-	30	32	24*
Maximum length of lower jaw	-	-	87*	96
Scapula total length	68r, 65l	64*r	73r	69*l

All lengths are in mm. Damage or incompleteness is marked with a * but in each case only a small amount of bone is considered missing. ‘r’ and ‘l’ denote right and left elements respectively where appropriate.

All four of the individuals are clearly immature animals. The sutures in the skulls have not fused fully and the orbits are large relative to the size of the skull, whereas the frills are proportionally small - all characteristic of juvenile *Protoceratops* [28], [29]. In A, C, and D, the respective scapulae and coracoids have not fused together and in C the ilia are not fused to the sacrum, and the neurocentral sutures in the caudals have not closed. Given the similarity of the dimensions of the various comparable elements, the four are presumed to have been similar ages.

Measurements of these specimens are comparable to those of a small *Protoceratops andrewsi* specimen reported by Brown and Schlaikjer [28], AMNH 6419 (American Museum of Natural History, New York, USA, scapula length  = 64 mm; midline skull length  = 130 mm). By contrast, they are approximately one-quarter the size of the largest reported *P. andrewsi* specimens (AMNH 6424, scapula length  = 231 mm; AMNH 6471, midline length  = 406 mm - American Museum of Natural History, New York, USA). However, these are considerably larger than the very young specimens from an assemblage of juvenile *Protoceratops* cf. *P. andrewsi* (MPC-D 100/530) described in [20] - maximum skull length here is ∼340% larger than the mean skull lengths reported for those young juveniles.

### Specimen MPC-D 100/534

**Figure 7:**
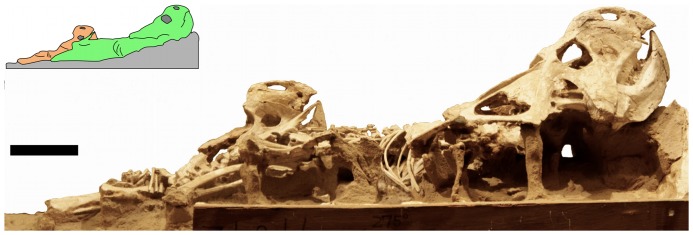
Specimen MPC-D 100/534 a pair of subadult *Protoceratops* in right lateral view. Inset shows the division between the specimens. Scale bar is 100 mm.

Specimen MPC-D 100/534 includes a nearly complete subadult *Protoceratops* (lacking only part of the tail) and a second poorly preserved subadult individual (represented by the premaxillae, maxillae, anterior part of the mandible, partial right limbs, and right sided dorsal ribs). The animals lie subparallel to one another (Figure 7), and are within a block of matrix approximately 1 m by 0.5 m. Their bodies were compressed, and the tilt of the skulls was slightly affected by compaction of the sands after burial. The tail on the more complete individual and the rest of the body on the second individual were destroyed by syndepositional erosion (sand dune migration).

In 1988 in the same area of sand beds of Tugrikin Shire [47], a team from the Hayashibara Museum Natural Science and Mongolian Paleontological Center Joint Paleontological Expedition also found the skull with articulated partial postcranial skeleton (articulated cervicals and cervical armour in situ) of the ankylosaur *Pinacosaurus*. At that time, the rest of the postcranial skeleton (girdles and other distal parts) were not exposed on the surface. However, the posterior part of the neck of the specimen showed a clear and sharp termination, as if that part had been cut and the remaining part had been separated from the skull (personal observation Watabe in field.) This phenomenon suggests that the syndepositional erosion had been occurred in eolian sedimentation of Tugrikin Shire, and during the recycling of the sands, the dinosaur skeletons (whole or part of them) were and damaged on the surface before reburial, and their subsequent discovery. This process is hypothesised to have also affected the animals of MPC-D 100/534.

As with the set of juveniles, there is some limited settling of the bones, and there is some damage to joints from insect larvae [44] and there is a possible mammal burrow (based on the size of the hollow) entering between the dorsal ribs of the better-preserved animal. The main specimen can be identified as a subadult (*sensu* 30) based on size as well as the development of the cranial ornamentation, which is distinct but lacks the presumed ultimate morphology seen in the largest specimens e.g., strongly developed nasal ornamentation and an erect frill.

**Figure 8:**
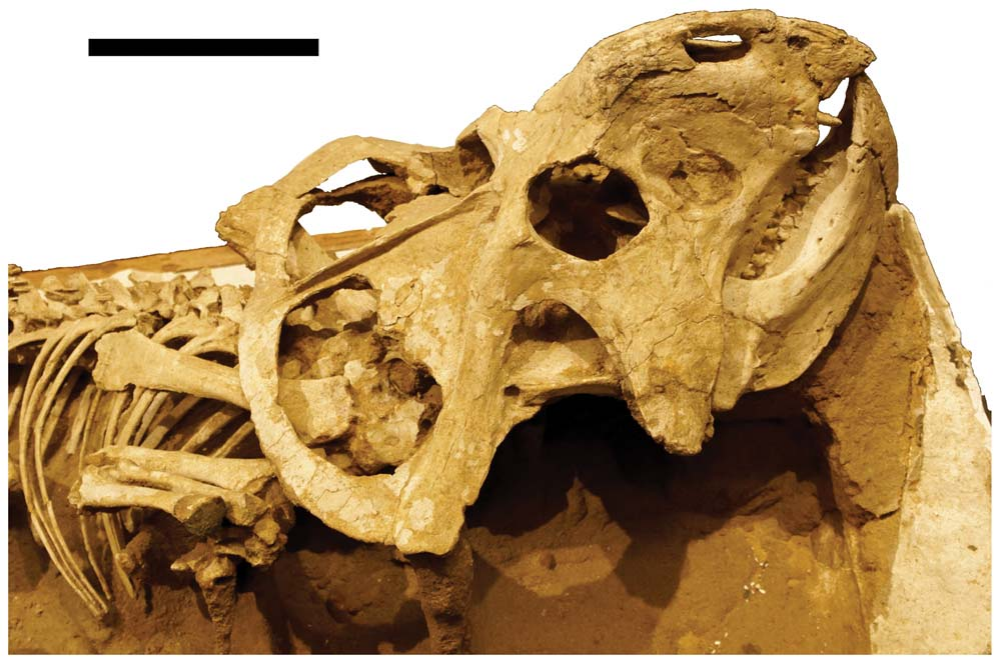
The better preserved skull of the MPC-D 100/534 pair in right lateral view. Scale bar is 100 mm.

The better preserved specimen of the subadult pair (Figure 8) has a total skull length of 310 mm (from tip of rostral bone to end of frill), a scapula measuring 134 mm in length, and femur of 123 mm (though both of the longbones are damaged at their articular ends and would have been a little larger in life) which are comparable in length to equivalent elements in other subadult *Protoceratops* (e.g., versus a 198 mm long scapula for CM 9185, Carnegie Museum of Natural History, formerly AMNH 6471; [28] - American Museum of Natural History, New York, USA). The second specimen appears to be a little larger, although little is preserved anatomical reference points can be identified and the tip of the snout to the anteroventral margin of the orbit is measured at 127 mm, which is larger than the same measure in the better preserved animal which is 118 mm.

### Identification

Identification of MPC-D 100/534 is easier than the juvenile specimens because the individuals are closer to adult status and thus exhibit more clearly the normal diagnostic features of the species. The subadult pair is also diagnostic to *Protoceratops andrewsi*, based on the broad fenestrated parietal with a tab-like process at the anterior end of the fenestra and the triangular rostral bone with straight posterior margin, among other features [30]. It is further differentiated from *Bagaceratops rozhdestvenskyi* by the lack of an accessory antorbital fenestra and from *B. rozhdestvenskyi* and *P. hellenkorhinus* by the presence of premaxillary teeth.

## Discussion

### Burial of the specimens

The aeolian deposit and monospecific assemblage suggest that in both cases the individuals died simultaneously, rather than accumulating as time-averaged assemblages. In the case of the juvenile assemblage, the posture and overlapping positions of the animals with the upturned heads strongly suggests that they were alive at the time of burial. The animals may not have been completely restricted in their movements, either (at least initially). This is consistent with other specimens from the same area [27].

Evidence for this hypothesis comes from the postures of the animals in MPC-D 100/526; all four animals are in a relatively normal life orientation (feet down, horizontal orientation of the axial column), and have not been turned at random or tumbled. Individuals B, C and D have upturned heads, and both B and C are in a position suggesting they are trying to move upwards, with the forelimbs at a level above the hind, and individual C even has its mouth open.

A number of *Protoceratops* specimens have been found buried in a similar posture, with the heads uppermost and the rest of the body angled downwards [27]. These have been interpreted as animals buried in a burrow whether it collapsed or infilled as a result of a sandstorm, although Jerzykiewicz et al. [27] specifically suggested this was not the case. In the situation here, if these young animals had been in a burrow together they would most likely have a similar orientation, with their long axes being subparallel to each other and at similar angles to the horizontal, because they would be restricted by the diameter and orientation of the burrow. Thus, we infer that the animals were above ground when the event took place. Clearly, a large amount of sand was deposited on the animals relatively quickly, though this could have come from an especially heavy sandstorm [27], or perhaps the collapse of a sand dune after rain [48]. In the case of the latter possibility, attempts to free themselves would have likely resulted in additional sand falling and eventually burying the animals.

Eventually the cover was heavy enough to bury the animals fully, preventing their escape, causing their death, and preserving their positions. Heavy cover would also restrict scavenging by vertebrates or the invasion of insect larvae hence the relative completeness and condition of the material. Later, decay of the soft tissues and general compaction allowed the sand to settle, causing the vertical displacement of some elements while the majority remained in articulation. In the case of the subadults (MPC-D 100/534 – Figures 7, 8), a similar position has been previously noted for an aggregation of adult *Protoceratops*. Jerzykiewicz [27] noted that the animals lay subparallel to one another, facing in the same direction, and this is also reminiscent of the arrangement seen in the hatchling group described by Fastovsky et al. [20].

Collectively, the evidence strongly suggests in both cases that these animals were together at the time of death. They were positioned very close to one another, yet no other specimens were found in the immediate vicinity. They would appear to be of near identical size and thus presumably ontogenetic stage, and it is considered likely that in each respective case they were part of the same cohort [22].

Such mass-mortality aggregations and groups of conspecifics preserved together have often been used to infer social behaviours in non-avian dinosaurs. However, in order to do so correctly, a better understanding of sociality in extant forms, definitions of sociality and the plasticity both within and between species is required.

### Inferring social behaviours in dinosaurs

The word “social” may be used as a catch-all term to refer to a group of animals that are spending time together and interacting, but this covers a myriad of behaviours and degrees of social interactions, hierarchies, dependence, and amounts of time spent in association. While ‘sociality’ is generally used to imply animals interacting and generally in close proximity, any interactions between conspecifics may be deemed social and can occur though calls or through olfactory cues (e.g. territory marking) such that the exchanges occur with such temporally or spatial separation that the animals involved do not actually meet.

When discussing evidence for sociality in the fossil record therefore, clarity is required with definition and distinctions must be made between sociality with respect to group living and intraspecific interactions. Care should also be taken to avoid conflating different terms [49] such as the aggregation of multiple individuals of a species as being considered evidence for as ‘sociality’ for a species as a whole. For example, evidence of structured intraspecific combat in *Triceratops* [50] does not necessarily mean that the animal habitually lived in groups [11]. Individuals may reside in a group only temporarily, or on a long-term basis but without interactions such that they represented merely a temporal aggregation with some social or socio-sexual interactions, as opposed to a habitual social and/or family group. Animals may congregate for any number of reasons, and there are wide ranges in levels of sociality [51] from being antisocial and aggressive to conspecifics, tolerating the presence of conspecifics under some circumstances, through to a conspecific group or social structure being critical to the individual's general well-being or even survival. These may also change with circumstance or during ontogeny and simply vary between individuals or populations.

For example among extant amniotes, at the extreme end of sociality, naked mole rats are obligate social animals and cannot survive except within a highly structured caste-based community [52]. Animals such as suricates (*Suricata suricata*) are also dedicated to a social system of co-operation based around small family groups [53]. Others such as cheetah (*Acinonyx jubatus*) are more plastic and can live socially or alone, and individuals may switch between the two [54]. The males of many social ungulates species (e.g. gnu – *Connochaetes taurinus*) may be solitary while females and juveniles live in family groups, or males may form ‘bachelor herds’ during adolescence, or when unable to control a territory or harem [55]. Many large herbivores form general loose aggregations of animals, but there may be more dedicated family groups or harems within a larger herd, though actual interactions, cooperation or sharing of resources may be nonexistent (i.e. they would best be described as gregarious without being strongly social). Finally there are species that, provided there is no direct threat or competition from conspecifics, may be equally content in aggregations or be solitary as the local environment or circumstances dictate (e.g. gharial – *Gavialis gangeticus* [56]).

It is worth noting that while sociality has been best been studied and described in birds and mammals, complex social/group behaviours have also been documented in squamates [57]–[59] and crocodilians [60]. As such, while the best examples may lie within mammalian and avian systems (likely in part as these have been more extensively studied to date), there is no reason to think that such behaviours were beyond the capabilities of non-avian dinosaurs, or that considerations of their behaviour should be strictly limited to phylogenetic bracketing (see also [61] on sociality in extant reptiles).

Social groups may form for a number of reasons, and sociality or group living may be highly variable both ontogenetically and phylogenetically. Bekoff et al. [62] note an important and interesting conflict of signals in extant carnivores for degrees of sociality where there may be strong plasticity in terms of intraspecific variation, but that there may still be a strong ‘norm’ for the taxon. Even so, among African and Asian felids for example, we see great variation in what is considered normal. Even among closely related taxa living in similar or identical environments and occupying the same feeding guilds (that is, they are feeding on similar species), there is a broad range of degrees of sociality. We see animals that are effectively solitary such as the tiger (*Panthera tigris* [63]) and leopard (*Pantera pardus* [64]), but also those where animals may hunt alone or in pairs such as the caracal (*Caracal caracal* [65]) or hunt in groups, pairs or alone such as the cheetah (*Acinonyx jubatus*) or lion (*Pantera leo*). In the case of the latter, lions are best known for hunting in social groups termed prides, typically consisting of one or two dominant males, a number of females and their offspring. However, adolescent and older males may become ‘nomads’ and hunt alone or in pairs between times of being part of a pride [66]. In the case of cheetah, adult females tend to be solitary and adult males tend to live in groups, while adolescents of either gender may live alone or in groups of siblings, and females may associate with males around the time of mating [54] (p. 7). Thus the cheetah represents an entire complex of degrees of sociality that may change from juvenile to adult and/or with gender – any given cheetah may be solitary or social and switch from one to the other. The felid example here is perhaps extreme or unusual (herbivores unlike carnivores are regularly at strong risk from predation and so may tend to aggregate more often than their predators) but it does demonstrate the range of social behaviours seen both inter- and intraspecifically.

Individuals or groups may be forced together or simply coalesce into large aggregations than they would normally owing to surrounding circumstances. Something as simple as a breeding season, or resting at night through to mass migrations will cause large aggregations to form even if the animal is more normally solitary. Poor conditions such as a drought may force animals to areas of remaining water or food (or perhaps limited nesting sites – see [67] for an especially interesting example), or alternatively plentiful resources can lower the barriers of competition such that anti-social animals can gather together in large numbers with greater tolerance such as seen with caiman for example [60]. Notably from the point of view of palaeontologists trying to make inferences about the life of extinct animals, such events as migrations could potentially lead to large numbers of individuals of one species dying together in a single location, even if those animals spent most of their time alone.

Collectively therefore, even with strong palaeontological evidence for members of a genus or species being together (e.g. through multiple monospecific aggregations that represent single mass-mortality events), it is difficult to make firm assertions about whether or not the taxon in question typically lived in groups, and whether or not these groups were social, given the variation from group to solitary living present in at least some species. Similarly, it is incorrect to assume that a mass mortality site with even dozens of individuals preserved will have a 50∶50 sex ratio or something close to it – a herd may be composed solely of males, or be a harem of many females with a single dominant male (e.g. Springbok and Thompson's gazelle [68] p. 82). Moreover, inferences from one taxon should not normally be extended to other taxa given the range of behaviours seen in even closely related taxa. Nevertheless, the regularity with which some dinosaurs are found in close associations, supplemented by trackways and evidence of social interactions and communication [50], [69]–[70] suggest that numerous dinosaurs spent at least some of their time in groups and by extension are strong candidates to be considered social animals. Given the rarity of non-selective, mass-mortality events that preserve monospecific bonebeds, such events must be considered excellent evidence for those animals being naturally aggregated at the time of death.

As such, we advocate a more rigorous approach to discussing putative sociality in dinosaurs. A simple monospecific aggregation of a taxon should not alone be considered evidence for sociality. We suggest that more specific terms be used for various possible degrees of sociality and most especially recognise the difference between a group that may be ‘social’ (here we mean in the sense that there are social interactions and also likely some form of social hierarchy and/or bonds between the members of the group), and those that are ‘gregarious’ (here we mean that animals are aggregating into a group that live together but lacks social interactions). Numerous definitions of degrees of sociality are present in the ethological literature (e.g. see [71] on varying definitions of ‘social’ and ‘eusocial’) and palaeontologists need to settle on one or more definitions that best allow them to describe the available material. However, we feel that the science is not best served by the current situation where multiple different terms seem to be used interchangeabl, y and without regard to definitions for those terms.

### Behaviour of juvenile dinosaurs

Even within the complexities and shifting degrees of sociality described above, a degree of sociality is seen in various juvenile amniotes, even when this is not seen in adults [58]. Although very young animals (especially nestmates) will often remain together shortly after leaving the nest or during parental care, they may continue to band together once independent of their parents. Bands may form with juveniles of the same cohort or even with individuals of different ages and live together in this manner for considerable periods of time, only leaving the group upon maturity. Crèches may also form when juveniles come together, and this may or may not be under the protection of one or more supervising adults [72] (p. 290–291) [73] (p. 194–195).

Predation is a major factor in driving group living [74] (p. 8–23) and crèche formation [72] (p 291 and references therein). Among metazoans, most juvenile deaths are through predation [75] with few individuals making it past their first or second year of life, and juvenile animals are especially vulnerable to predators for a variety of reasons. One major issue of juvenile vulnerability is through a combination of factors that reduce vigilance – they generally require lots of resources as they are growing thus necessitating long foraging times, they forage in poor quality areas (increasing foraging time further, and increasing risk of ambush) and are inexperienced foragers necessitating still longer foraging times ([76] and references therein). Because of the dangers posed by juveniles to adults (they can attract predators), in groups that do not exhibit extended parental care or highly structured social systems, juveniles may be excluded from social groups and aggregations. Thus juveniles are left to fend for themselves and without the benefit of the protection or experience that could be offered by adults.

However, a number of benefits are provided by living in groups ([77] p. 472, [78] p. 175, [79]). Most notably, vigilance can be increased by banding together: each individual can reduce the amount of time expended in vigilance [80]–[81] but collective vigilance will be increased and so by extension increased defence against predation. There is also the bonus of increasing the numbers in the group such that any attack will less likely strike any given individual (the ‘dilution effect’ [74] p. 13, [73] p. 362–364]. Finally, by coming together they become harder to find as they are less evenly distributed across the environment but instead become patchy as a resource (for a predator).

Although there is evidence for some post hatching parental care in numerous lineages of dinosaurs, there is only limited evidence for care beyond the earliest parts of the lives of a given nest of animals [82]–[83]. As noted above, although there is evidence of mixed social groups containing juveniles and adults [4], most records of groups of dinosaurs are composed solely of non-adult animals, or adults alone [10]. Young juveniles would apparently be left to fend for themselves from a relatively early age, and banding together would be an obvious form of defence.

The specimen of juveniles described here is inferred as evidence for this general hypothesis, and indeed the majority of juvenile aggregations of dinosaurs support this contention. The mixture of sizes of specimens in juvenile aggregations of animals that are not nestlings or hatchlings [17] suggests that juveniles from those groups were not just siblings from one nest (as further supported by the number of individuals present and the small chances of all hatchlings of a single nest surviving for so long given typical infant mortality inferred for dinosaurs [76]). They were probably not simply a group of nestmates staying together for extended periods but a group composed of individuals from multiple nests of one cohort and perhaps from different parts of the breeding season or even from different years. Note that such aggregations do not necessarily follow that adults of the same species will necessarily be sociable: adults with the benefits of experience, reduced foraging times, larger size, and perhaps also the addition of fully developed armour or weapons (e.g. horns, osteoderms) would not be so vulnerable to predators.

It is also worth noting that young and inexperienced animals are also perhaps vulnerable to making mistakes that adults might not. While there are records of entire assemblages of adults that have died in some natural disaster or extreme conditions (e.g. *Shantungosaurus* [84]) it is notable that at least some juvenile groups have died in more ‘mundane’ and perhaps avoidable situations such as mudtraps [5]–[6], [10] and may simply be trapped because of their size – adults might be able to wade through waters that would drown juveniles for example.

Although juveniles are taphonomically less likely to be preserved that adults animals (in addition to removal through predation), we now have numerous examples of juveniles banding together beyond the age for which we have evidence of parental care [5], [17] and in the absence of adults. There are also groups of adults preserved without the presence of juveniles. Significantly, there are specimens such as the one presented here and that of Zhao et al. [7] that seem to have been overwhelmed instantly or very rapidly. As such, were adults present with the group, we would expect them to be preserved as they would not have escaped, unlike (potentially) a mudtrap where a large adult might escape [5], or might have the experience to have avoided danger.

Clearly each fossil aggregation of dinosaurs must be assessed on a case-by-case basis and take factors such as taphonomy into account. Animals may aggregate in life to breed or migrate for example, or even simply frequent a location (e.g. following a game trail, or going to a watering hole or mineral lick) and thus leave evidence of large numbers of individuals together (eggs, nests, trackways or even mass mortality sites) without them necessarily being fundamentally sociable. However, the numerous examples of exclusively juvenile aggregations in the fossil record must suggest that juveniles of at least some taxa were often gregarious. Moreover, at least some specimens are preserved in a manner suggesting that the group genuinely was living together and was not simply an aggregation of bones or a group formed during migration etc., or numerous aggregations are known for a single taxon such that these cannot represent an unusual case. Based on analogy with the behavioural patterns of extant amniotes and the hypothesized vulnerability of juvenile dinosaurs to predators [76], we suggest that juveniles would aggregate primarily as a defence against predators and that some adults groups might have actively shunned juveniles from joining them, or may themselves have been solitary.

Group living does not necessarily bring benefits alone: although foraging efficiency may be increased by group living ([74] p. 23–25), since any individual might find a resource than can be exploited by the whole group, there will also be increased competition for local resources ([78] p. 176). Ultimately, gregariousness will occur only if on balance the trade-off for increased protection is greater than any losses through competition (and of course other factors on both sides of the equation). For example among long-tailed macaques (*Macaca fasicularis*), group size is notably smaller where predators are absent, showing that increased protection is an important (but not the sole) determining factor in group living [79]. Naturally though, the effects will be different for juveniles and adults because they face different pressures (juveniles need to grow but not reproduce, adults the opposite) hence why they may adopt different strategies. An adult may need to acquire or defend a specific resource be it food, territory or nesting site, or indeed a harem of mates. Simply having sufficient experience to forage successfully and avoid predators (or being of sufficient size or potency to ward them off) may mean an adult would on balance do better to forage alone in high-quality areas rather than face increase competition from a group if the levels of increased protection are low.

### Gregariousness in Protoceratops

It is perhaps significant that very young juveniles [20], mid-sized juveniles (MPC-D 100/526), subadults (MPC-D 100/534), and adult [27] *Protoceratops* are all now known in aggregations. As noted above, in at least some, and possibly many, dinosaurian taxa, a shift in the degree of sociality may be expected with juveniles aggregating, whereas adults might be solitary. However, based on the available material it appears that *Protoceratops* formed aggregations throughout their lives. Clearly these would vary in composition – siblings from one nests or local members of the cohort might aggregate together shortly after hatching, but losses through predation and other factors would reduce the numbers and smaller groups or perhaps individuals would aggregate into larger groups to increase its defence.

Notably, Fastovsky et al. [20] suggested that the assemblage of very young animals that they described was unlikely to be a “kind of aggregate of individuals, perhaps brought together during a sandstorm. If such were the case, it would be unlikely that the individuals be exclusively juveniles of the same age”. As noted above, our specimen consists of animals considerably older than those described by Fastovsky et al. [20] and yet of very similar sizes to one another, and the fact that a second group of juveniles alone is now known, reduces the impact of their assessment of this being ‘unlikely’ for the inferred hatchling group to be independent. The subparallel orientation of the animals in the hatchling aggregate is also seen in aggregates of adult *Protoceratops* [27]. As such, although we agree that the most likely interpretation for the Fastovsky et al. [20] aggregation is that these animals are nestmates, that they were an independently living social group rather than in a nest, cannot be ruled out and should be considered a plausible alternative hypothesis.

**Figure 9:**
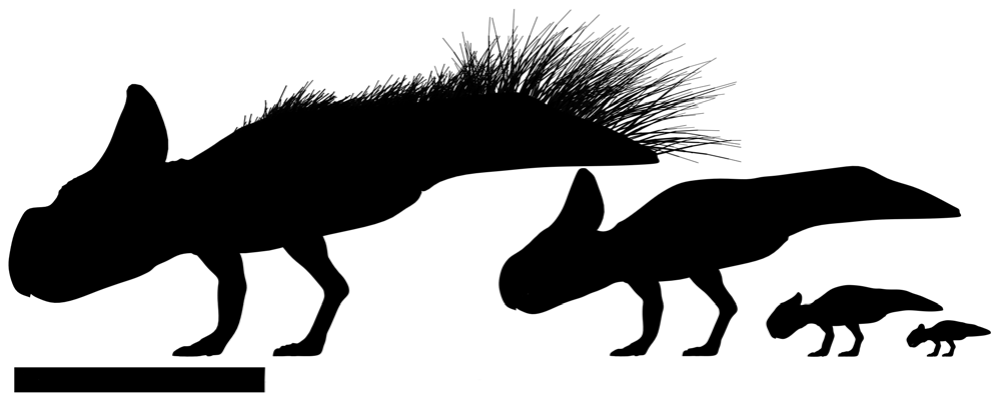
Sequence of silhouettes to illustrate the differing sizes of *Protoceratops* specimens considered here. Left to right: Adult animal based on data in [29], subadult animal based on MPC-D 100/534, midsized juvenile based on MPC-D 100/526, and finally a very small juvenile based on data from [20]. Scale bar is 1 m.

*Protoceratops* is the first non-avian dinosaur that provides evidence for the formation of single cohort aggregations throughout their life, from hatchlings to adult. Between the specimens described here and those elsewhere in the literature, young juveniles, mid-sized juveniles, subadults and adults are all now known in aggregations (Figure 9). The plasticity and variation of sociality in extant amniotes means that no absolute inferences can be made. While we cannot rule out the possibility that some individuals were primarily solitary, the available evidence is that restricted-age aggregations were likely a normal part of life for many *Protoceratops*.

*P. andrewsi* is known from a number of east Asian localities including both the Mongolian Tugrikin Shire locality, but also the Chinese Bayan Mandahu. The two formations have been considered to be both contemporaneous and non-contemporaneous [85], but in either case differences in the faunal compositions between the two localities and a geographic separation suggests that the *P. andrewsi* in each represented different populations. Aggregations of adults are known from both localities [27], though to date aggregations of juveniles are restricted to Tugrikin Shire. Isolated individual adults and non-adults are also known at both sites (DWEH pers obs. at Bayan Mandahu, and unnumbered specimens at the IVPP (Institute of Vertebrate Paleontology and Paleoanthropology, Beijing, China) and WM pers obs. and unnumbered specimens at the HMNS). As a result, the most conservative interpretation of the data currently available is that there is evidence only for gregariousness across ontogeny for the Tugrikin Shire population of *P. andrewsi*.

Although we cannot exclude the possibility that *Protoceratops* were social (spending much of their lives together, with associated social interactions and dominance hierarchies), we prefer to be more conservative and consider these data instead as evidence for gregariousness (a tendency to form groups, with no explicit inference of dominance or social interactions). Given the evidence for gregariousness at multiple life stages within *Protoceratops*, evidence for communal nesting [43] and sociosexual signaling structures [14], [70], we suggest that the Tugrikin Shire population of *Protoceratops* may have had social behaviours throughout ontogeny.

Overall though, this pattern may not be uncommon in the Dinosauria. The population structure of vertebrates is such that there tend to be few late stage juveniles or subadults at any given time (see [76] and references therein), and because entire aggregations of animals are rarely preserved these are perhaps less likely to be recovered than either collections of juveniles or adults. The very large numbers of *Protoceratops* recovered and the fact that it appears that numerous individuals died in dramatic events (which could trap entire groups, but also provided exceptional preservation conditions) may simply mean that this was the genus with the best chance of preserving a record of such behaviour, rather than it necessarily being rare.
